# Smartphone Dependence, Sleep Quality, and Suicidal Ideation Across Adolescence: A Six-Year Longitudinal Study

**DOI:** 10.3390/bs16101828

**Published:** 2026-10-06

**Authors:** Yeonju Lee, Sung-Man Bae

**Affiliations:** 1 Department of Counseling Psychology, Graduate School, Kyungnam University, Changwon 51767, Republic of Korea; zhzm7@kyungnam.ac.kr; 2 Department of Psychology, College of Health Science, Dankook University, Cheonan 31116, Republic of Korea

**Keywords:** suicidal ideation, smartphone dependence, sleep quality, adolescents, COVID-19

## Abstract

Suicide is a leading cause of death during adolescence, and both smartphone dependence and poor sleep have been associated with suicidal ideation. However, it remains unclear whether these associations reflect stable differences between adolescents or year-to-year changes within adolescents. This study examined the developmental trajectories of smartphone dependence, sleep quality, and suicidal ideation over six years spanning the onset of the COVID-19 pandemic and tested whether sleep quality accounted for the association between smartphone dependence and suicidal ideation at the between-person and within-person levels. Data were drawn from six annual waves (2018 to 2023) of the Korean Children and Youth Panel Survey 2018 middle school first-grade cohort (*N* = 2590; 54.2% boys). A parallel piecewise latent growth curve model (LGCM) and random-intercept cross-lagged panel model (RI-CLPM) were estimated. Smartphone dependence increased before the pandemic onset (*B* = 1.47 per year) and declined slightly thereafter (B = −0.16), sleep quality declined after the onset (*B* = −0.05), and suicidal ideation decreased before the onset (*B* = −0.10) and increased gradually thereafter (*B* = 0.03), with no immediate shift at the onset. In the LGCM, the indirect effect of smartphone dependence on suicidal ideation through sleep quality was significant for usual levels (*B* = 0.005, *p* < 0.001) and pre-onset change (*B* = 0.008, *p* = 0.001) but not for post-onset change (*B* = −0.001, *p* = 0.570); Wald tests indicated that the path from sleep quality to suicidal ideation (*p* = 0.001) and the indirect effect (*p* = 0.004) differed between the two periods. In the RI-CLPM, higher-than-usual smartphone dependence predicted higher-than-usual suicidal ideation one year later (β = 0.05, *p* < 0.001), whereas no within-person lagged paths involving sleep quality were significant. Smartphone dependence, poor sleep quality, and suicidal ideation thus co-occurred between adolescents, but annual within-person changes did not show a pathway through sleep quality. Because period differences cannot be separated from age-related development in a single cohort, they should not be interpreted as pandemic effects. These findings support attending to adolescents with high smartphone dependence and poor sleep within existing school-based screening systems, whereas the mechanisms through which risk is transmitted require studies with shorter measurement intervals.

## 1. Introduction

Suicidal ideation is an important concern in adolescent mental health, particularly in South Korea, where suicide is the leading cause of death among adolescents (K. Lee et al., 2025; Statista, 2024). Globally, suicide is the third leading cause of death among people aged 15–29 years (World Health Organization, 2025). In 2023, the suicide rate among Korean adolescents aged 10–19 years was 7.9 deaths per 100,000, a 10.4% increase from the previous year (Ministry of Health and Welfare, 2024), whereas youth suicide trends elsewhere have varied, with declines reported in many European countries (Bertuccio et al., 2024). The ideation-to-action framework distinguishes the development of suicidal ideation from the progression from ideation to suicidal behavior; these processes may have partly different correlates (Klonsky & May, 2015; Nock et al., 2013). In a nationally representative US sample, approximately one-third of adolescents with lifetime suicidal ideation had made a suicide attempt (Nock et al., 2013). Among adolescent psychiatric inpatients, persistently elevated suicidal ideation was associated with subsequent attempts (Czyz & King, 2015). Nevertheless, individual risk factors have limited predictive accuracy (Franklin et al., 2017). Longitudinal examination of potentially modifiable correlates of suicidal ideation can inform questions for prevention research, but observed associations alone cannot justify screening or establish the effects of intervention.

Smartphone dependence is one potentially modifiable correlate. In South Korea, the proportion of adolescents classified as being at risk of smartphone overdependence was 43.0% in 2025, the highest of all age groups, even as the proportion among all smartphone users declined (Ministry of Science and ICT, 2026). Smartphone dependence refers to problematic use involving difficulty controlling use, psychological reliance, and interference with daily functioning (Billieux, 2012). Whether it constitutes a behavioral addiction remains debated (Panova & Carbonell, 2018). Problematic smartphone use has been associated with depression, anxiety, stress, and poor sleep quality (Elhai et al., 2017; Sohn et al., 2019), and in a two-wave study of Chinese high school students, mobile phone addiction predicted suicidality over the following five months (Li et al., 2022). In a large US cohort, high or increasing trajectories of addictive mobile phone and social media use were associated with suicidal ideation and behavior, whereas total screen time measured at baseline was not (Xiao et al., 2025). These findings motivate attention to patterns of problematic use across adolescence alongside the amount of time spent using devices.

Perceived sleep quality is a plausible correlate of both smartphone dependence and suicidal ideation. A meta-analysis of adolescent studies found associations between sleep disturbances and suicidal ideation in cross-sectional studies, as well as evidence of prospective associations (J.-W. Liu et al., 2019). In South Korea, academic schedules may constrain sleep duration; a national survey conducted in 2020 reported an average of 6.3 h of sleep and found that 30.8% of adolescents were satisfied with their sleep (D. H. Kim et al., 2022). Perceived sleep quality captures aspects of sleep that duration alone may not reflect, although a single self-report item cannot measure its distinct components. Sleep problems may be linked to suicidal ideation through difficulties in emotion regulation (Palmer & Alfano, 2017). An intensive monitoring study among adolescents and young adults at high risk for suicide found associations between poorer sleep and next-day suicidal ideation involving affective reactivity to interpersonal events (Hamilton et al., 2023). Smartphone use may also be related to sleep: a theoretical review suggested that device use beyond the intended bedtime may displace sleep, while the experimental evidence for effects of screen light and stimulating content on sleep onset indicated relatively small effects (Bauducco et al., 2024). A meta-analysis also reported an association between greater smartphone use and poorer self-reported sleep quality (Chu et al., 2023). The direction may be reciprocal; sleep difficulties may precede technology use, for example when adolescents use devices before falling asleep (Bauducco et al., 2024). In a three-wave analysis of the same Korean panel used here, poorer perceived sleep quality predicted later smartphone overdependence, whereas the reverse path was not supported (Oh & Heo, 2026). The direction of the association should therefore be examined empirically.

Despite these findings, it remains unclear whether associations among smartphone dependence, perceived sleep quality, and suicidal ideation are found in adolescents’ usual levels, their developmental trajectories, and their year-to-year deviations from usual levels. Previous adolescent studies have not consistently examined all three constructs together across multiple years; a recent three-wave analysis of the same Korean panel, for example, examined depressive symptoms rather than suicidal ideation (Oh & Heo, 2026). In addition, conventional cross-lagged panel models do not distinguish stable between-person differences from within-person deviations, which can lead to different interpretations of longitudinal associations (Hamaker et al., 2015). Associations between adolescents do not necessarily describe what happens when an individual adolescent’s smartphone dependence or sleep quality changes. Studies with intensive monitoring have examined sleep and suicidal ideation at shorter intervals (Hamilton et al., 2023), but those results cannot establish whether a sleep-related pathway is present at annual intervals in a community-based adolescent cohort. Distinguishing these associations can clarify what the available panel data show; it cannot, by itself, establish whom to screen or when an intervention would be effective.

The six-year observation period also spanned the onset of the COVID-19 pandemic, a period during which adolescents’ school routines and opportunities for in-person interaction changed. Korean survey data suggest that changes in sleep and mental health were not uniform across the pandemic years (D. H. Kim et al., 2022; Y. Kim et al., 2025; J. Lee et al., 2022). Because the pandemic coincided with age-related change in this single cohort, differences between periods before and after its onset cannot be attributed to the pandemic itself. We therefore examined differences across these periods without treating them as estimates of a pandemic effect.

Using six annual waves (2018–2023) of the Korean Children and Youth Panel Survey 2018 (KCYPS 2018; National Youth Policy Institute, 2019), this study had three aims. First, we described the developmental trajectories of smartphone dependence, perceived sleep quality, and suicidal ideation across adolescence. Second, we examined whether associations among usual levels and rates of change in these variables were consistent with an indirect pathway through sleep quality, and whether the estimated growth-factor associations differed between the periods before and after the pandemic onset. Third, we examined within-person associations across annual waves, including whether the hypothesized lagged pathway through sleep quality was supported. We used a parallel piecewise latent growth curve model (LGCM) for the first two aims and a random-intercept cross-lagged panel model (RI-CLPM) for the third.

## 2. Materials and Methods

### 2.1. Participants

This study used raw data from the KCYPS 2018 conducted by the National Youth Policy Institute (2019). KCYPS 2018 is a large-scale national longitudinal panel survey that employs a stratified multistage cluster sampling design to select a nationally representative sample and follows the same participants annually.

The present study focused on the middle school first-grade cohort of KCYPS 2018 and used data from six annual waves (2018 to 2023), covering the developmental period from the first year of middle school through the third year of high school. The surveys were conducted between August and November of each year (August to December in 2020), with trained investigators visiting each household to conduct in-person surveys. In 2020, the third wave was conducted using a combination of in-person and online methods because of the COVID-19 pandemic.

In 2018, the first year of the survey, the original panel comprised 2590 children (boys = 1405; girls = 1185) selected by stratified multistage cluster sampling. In each subsequent year, the number of children who participated in the survey was 2438 in 2019, 2325 in 2020, 2160 in 2021, 2052 in 2022, and 1975 in 2023, corresponding to a retention rate of 76.3% at the sixth wave.

The 76.3% retention rate reflects the KCYPS 2018 official participation count, which includes only participants who completed the survey in full. However, the availability of data on the three main outcome variables was higher, as some partial participants provided valid responses on these specific measures. At Wave 6, valid data on at least one of the three main outcomes were available for 2224 participants (85.9% of the initial sample), and the missing data rate for each main outcome variable at Wave 6 was approximately 14%. To evaluate whether selective attrition might bias the results, we compared participants with valid data on at least one main outcome at Wave 6 (*n* = 2224) with those without any Wave 6 data on the main outcomes (*n* = 366) on their Wave 1 characteristics. The two groups did not differ significantly on Wave 1 smartphone dependence (*t* = 0.78, *p* = 0.438, Cohen’s d = 0.044), Wave 1 sleep quality (*t* = −1.38, *p* = 0.169, Cohen’s d = −0.074), or Wave 1 suicidal ideation (*t* = 1.17, *p* = 0.244, Cohen’s d = 0.065). A small but statistically significant difference was observed in sex distribution (χ^2^(1) = 4.61, *p* = 0.032), with a slightly lower proportion of girls among those who dropped out (40.4% vs. 46.6%, among retained participants); however, the effect size was very small (Cramér’s V = 0.042), and sex was included as a covariate in all subsequent models. Detailed attrition analysis results are provided in Supplementary Table S2. Although no baseline differences were found on the three outcome variables, this comparison cannot rule out attrition related to unobserved values at later waves.

The inclusion criteria for this study were participation in the middle school first-grade cohort of KCYPS 2018 and availability of data for at least one survey wave during the observation period. On this basis, the final analytic sample was defined as the original 2590 participants from the first wave. The present analysis focused on three variables (smartphone dependence, sleep quality, and suicidal ideation) that were assessed across six annual waves.

### 2.2. Procedures

This study used KCYPS 2018 data across six annual waves, from the first wave (2018) through the sixth wave (2023). Because the third wave (2020) coincided with the COVID-19 pandemic, the pandemic period was used as a reference point to distinguish changes in the outcome variables before and after COVID-19. Specifically, the first and second waves (2018–2019) were classified as the pre-COVID period and the third through sixth waves (2020–2023) as the post-COVID period, with the third wave serving as the transition point. In the piecewise LGCM, the third wave functioned as the knot shared by the pre-COVID slope (Waves 1–3) and the post-COVID slope (Waves 3–6); in the RI-CLPM, the third through sixth waves constituted the post-COVID period for the mean-level shift.

This study was approved for exemption by the Institutional Review Board of Dankook University (IRB No. 2026-03-013). The data used publicly available de-identified secondary data, and the researchers conducted the analysis using openly released data. All research procedures were conducted in accordance with the guidelines and regulations of the Institutional Review Board.

### 2.3. Measures

#### 2.3.1. Smartphone Dependence

Smartphone dependence was assessed using the 15-item Smartphone Addiction Proneness Scale for Adults (SAPS; D.-I. Kim et al., 2012). The SAPS was originally developed within a broader national project by the National Information Society Agency of Korea that produced parallel versions for both adolescents and adults, sharing the same theoretical framework of four sub-domains: disturbance of adaptive functions, virtual life orientation, withdrawal, and tolerance (D.-I. Kim et al., 2012; D. Kim et al., 2014). A subsequent adolescent version validated on a nationally representative sample of 795 Korean elementary, middle, and high school students confirmed the same four-factor structure with high reliability (D. Kim et al., 2014), supporting the applicability of these items to adolescents. Each item was rated on a four-point Likert scale. Higher scores indicate higher levels of smartphone dependence. Cronbach’s α values for the six annual waves ranged from 0.823 to 0.878 (T1 = 0.876, T2 = 0.868, T3 = 0.878, T4 = 0.823, T5 = 0.853, T6 = 0.851), indicating acceptable internal consistency.

#### 2.3.2. Sleep Quality

Sleep quality was assessed using an item on daily living time collected in KCYPS 2018. The item was originally adapted from the 2013 Survey on the Utilization of Youth Media and modified to evaluate subjective sleep status along with bedtime and wake-up time (National Youth Policy Institute, 2019). The item asked, “How well do you usually sleep?” and was rated on a four-point Likert scale, ranging from 1 (“very poorly”) to 4 (“very well”). Higher scores indicate better sleep quality. Because KCYPS 2018 assessed sleep quality with a single item, our indicator inherits this measurement constraint. A one-item measure cannot distinguish domain-specific components of sleep such as latency or night awakenings, nor permit conventional internal consistency estimation. Its broader implications for interpretation are addressed in the Discussion (Section 4).

#### 2.3.3. Suicidal Ideation

Suicidal ideation was measured using an item derived from the Symptom Checklist-90-Revised (SCL-90-R) (Derogatis, 1994) Korean version (K. I. Kim et al., 1984), adapted from an item used in the Korean Children and Youth Panel Survey (KCYPS 2010) (National Youth Policy Institute, 2010). The item asked, “I have thoughts of wanting to die,” and was rated on a four-point Likert scale (1 = strongly disagree, 2 = somewhat disagree, 3 = somewhat agree, 4 = strongly agree). Higher scores indicate higher levels of suicidal ideation. As with sleep quality, suicidal ideation was assessed using a single item in KCYPS 2018. This item does not distinguish the frequency, duration, or intensity of suicidal thoughts, and its internal consistency cannot be estimated; the implications for interpreting the findings are discussed in the Discussion (Section 4).

### 2.4. Statistical Analysis

To test the hypotheses of the present study, the primary structural models and descriptive statistics were estimated using Mplus version 6.12 (Muthén & Muthén, Los Angeles, CA, USA; Muthén & Muthén, 2010), and supplementary analyses conducted during revision were estimated using Mplus version 8 (Muthén & Muthén, 2017). Longitudinal measurement invariance was tested in R (version 4.5.2; R Foundation for Statistical Computing, Vienna, Austria) using the lavaan (version 0.7.2; Rosseel, 2012) and semTools (version 0.5.9; Jorgensen et al., 2026) packages, and the attrition analysis was conducted in Python (version 3.12.3; Python Software Foundation, Wilmington, DE, USA) using the pandas (version 3.0.2) and SciPy libraries (version 1.17.1). First, descriptive statistics were computed by calculating the means and standard deviations of smartphone dependence, sleep quality, and suicidal ideation, and bivariate correlations were examined. Given the inevitable occurrence of missing data across the six annual waves of the panel survey, missing values were assumed to be missing at random and were handled using full-information maximum likelihood (FIML) estimation, which makes use of all available information (Enders, 2022). The FIML estimator provides unbiased parameter estimates under the MAR assumption and is generally recommended over listwise deletion in longitudinal analyses (Enders, 2022). An attrition analysis (Section 2.1) showed no baseline differences between retained and non-retained participants on the three outcome variables, and sex, which was associated with attrition, was included as a covariate in all models. These results are consistent with, but cannot confirm, the MAR assumption.

Prior to the main longitudinal analyses, longitudinal measurement invariance of the smartphone dependence scale was examined at the item level across the six annual waves to determine whether changes over time reflected genuine developmental change rather than changes in measurement properties (Meredith, 1993). A four-factor measurement model comprising disturbance of adaptive functions, virtual life orientation, withdrawal, and tolerance was estimated using the mean and variance adjusted weighted least squares (WLSMV) estimator, given the ordinal nature of the items. Configural, metric, and scalar invariance models were then evaluated sequentially. Invariance was assessed based on changes in model fit, with ΔCFI ≤ 0.010 and ΔRMSEA ≤ 0.015 indicating that the additional equality constraints did not meaningfully worsen model fit (F. F. Chen, 2007). If full measurement invariance was not supported, partial invariance was examined by releasing selected equality constraints based on modification indices and substantive considerations (Byrne et al., 1989).

To examine how smartphone dependence, sleep quality, and suicidal ideation changed over time during adolescence, latent growth curve modeling (LGCM) was employed (Bollen & Curran, 2005). Given the potential impact of the COVID-19 pandemic, a piecewise LGCM was specified with the third wave (2020) as the point of transition. This model allowed for the simultaneous estimation of slope factors reflecting change before and after the pandemic, as well as a step factor capturing the abrupt shift in mean level at the onset of the pandemic. In addition, a parallel-process LGCM, including smartphone dependence, sleep quality, and suicidal ideation in the same model, was estimated to test associations among the intercepts and slopes of the three variables, addressing the first and second aims of the study.

The third wave (2020) was selected as the breakpoint because it coincided with the onset of the COVID-19 pandemic in Korea, when nationwide school closures were implemented in March 2020, remote learning was widely adopted, and social distancing measures were enforced. These abrupt environmental changes fundamentally altered adolescents’ daily lives and provided theoretical grounds for treating this wave as the developmental transition point. Because the third wave was collected between August and December 2020, at least five months after the school closures began, Wave 3 responses reflect adolescents’ circumstances during the pandemic rather than before its onset. Although in-person activities gradually resumed from 2022 onward, the initial pandemic onset represented the most abrupt disruption. To evaluate the robustness of this specification, a sensitivity analysis was conducted by comparing the primary piecewise model with a single-slope parallel process LGCM that assumed constant change across the six-year period without any breakpoint or step factor.

To formally test whether the mediation coefficients differed between the pre- and post-COVID periods, Wald tests were conducted within the primary piecewise LGCM using Mplus’s MODEL CONSTRAINT and MODEL TEST commands. Pre-COVID versus post-COVID differences were computed for the first-stage path (SDep → SQ), the second-stage path (SQ → SI), the direct effect (SDep → SI), the indirect effect (SDep → SQ → SI), and the total effect. A joint Wald test evaluated the null hypothesis that both the a and b paths were equal across the two periods. Wald tests were used because they are compatible with the MLR estimator applied in the primary model and allow formal coefficient comparisons without re-estimating the model.

As a robustness check for the treatment of sex as an intercept-only covariate in the primary model, the parallel piecewise LGCM was re-estimated separately for male (*n* = 1405) and female (*n* = 1185) subsamples. Sex differences in growth factor means, mediation paths, and indirect effects were evaluated using the z-test for the equality of regression coefficients (Paternoster et al., 1998), computed as $z=\frac{\left(B_{M}-B_{F}\right)}{\sqrt{\begin{array}{c} SE_{M^{2}}+SE_{F^{2}} \end{array}}}\;.$ Detailed results are provided in Supplementary Table S3.

To further examine the temporal ordering among the variables and within-person change, a random-intercept cross-lagged panel model (RI-CLPM) was estimated (Hamaker et al., 2015). The RI-CLPM decomposes each variable into a stable between-person random intercept and time-specific within-person deviations, thereby allowing the estimation of autoregressive and cross-lagged effects based on within-person variation. Using this model, the present study examined how smartphone dependence at a given time point predicted subsequent changes in sleep quality and suicidal ideation after controlling for individuals’ average levels, and how deviations in sleep quality in turn predicted subsequent suicidal ideation, thus addressing the third aim of the study. For parsimony and estimation stability, autoregressive and cross-lagged paths were constrained to be time-invariant (Mulder & Hamaker, 2021). Except for the measurement invariance models, which were estimated using WLSMV, all structural models were estimated using maximum likelihood with robust standard errors (MLR) to account for non-normality of the data, and model fit was evaluated using the criteria recommended by Hu and Bentler (1999) (comparative fit index [CFI]/Tucker–Lewis index [TLI] ≥ 0.95, root mean square error of approximation [RMSEA] ≤ 0.06, standardized root mean square residual [SRMR] ≤ 0.08). For complex longitudinal models, CFI/TLI ≥ 0.90 and RMSEA ≤ 0.08 were additionally considered acceptable.

## 3. Results

### 3.1. Descriptive Statistics and Correlations

The means, standard deviations, and correlation coefficients for the 18 variables included in the study are presented in Table 1. Descriptive statistics and correlations were computed using MLR estimation with the TYPE = BASIC option in Mplus 6.12, and missing data were handled using FIML. Coverage for all bivariate pairs was greater than 0.81 (*N* = 2590).

The mean of smartphone dependence increased from T1 (*M* = 30.58, SD = 7.32) to T2 (*M* = 32.05, *SD* = 7.06) and remained at approximately the low 32-point range through T6. Sleep quality showed little change across T1 through T3 (3.14 to 3.15) and then gradually declined from T4 onward, reaching 3.01 at T5 and 3.02 at T6. Suicidal ideation ranged from 1.52 to 1.58 across all waves, with only small differences in mean level across time points.

Within-construct temporal stability was positive and significant for all three variables. Across-wave correlations ranged from r = 0.21 to 0.58 for smartphone dependence, r = 0.09 to 0.42 for sleep quality, and r = 0.05 to 0.31 for suicidal ideation, with most correlations significant at *p* < 0.001. All three variables displayed a simplex pattern in which correlations decreased as the interval between measurements increased, indicating a data structure well suited to the subsequent longitudinal change models.

With respect to within-wave correlations, smartphone dependence was consistently negatively correlated with sleep quality (*r* = −0.17 to −0.05) and positively correlated with suicidal ideation (*r* = 0.18 to 0.30). Sleep quality and suicidal ideation were negatively correlated at most waves (*r* = −0.21 to −0.06). Cross-construct, cross-wave correlations showed a similar pattern of associations. Higher smartphone dependence was associated with lower subsequent sleep quality (*r* = −0.15 to −0.02) and higher subsequent suicidal ideation (*r* = 0.04 to 0.30), and lower sleep quality was associated with higher subsequent suicidal ideation (*r* = −0.17 to −0.04).

### 3.2. Measurement Invariance of the Smartphone Dependence Scale

Longitudinal measurement invariance results are presented in Table 2. The configural model, in which the four-factor structure was constrained to be identical across the six waves while factor loadings and thresholds were freely estimated, showed acceptable fit (CFI = 0.913, TLI = 0.901, RMSEA = 0.040, SRMR = 0.048), supporting the same factor structure across waves. The full metric model, which additionally constrained all factor loadings to equality across waves, showed a substantial decrement in fit relative to the configural model (ΔCFI = −0.054, ΔRMSEA = +0.010), indicating that full metric invariance was not tenable. Inspection of modification indices revealed that six items (items 5, 9, 10, 12, 13, and 15) showed loading deviations only at Wave 4. When these loadings were freed at Wave 4 only, the resulting partial metric model showed fit essentially equivalent to the configural model (ΔCFI = +0.002, ΔRMSEA = −0.001), supporting partial metric invariance. The partial scalar model, which additionally constrained item thresholds to equality while retaining the six freed loadings at Wave 4, showed a change in fit within the acceptable range relative to the partial metric model (ΔCFI = −0.004, ΔRMSEA = +0.001), supporting partial scalar invariance. Following the criteria of F. F. Chen (2007), these results indicate partial measurement invariance across the six annual waves. Because the structural models used sum scores, this finding supports the comparability of the scale across waves rather than directly justifying comparisons of latent means, and the deviations at Wave 4 should be considered when interpreting change around that wave.

### 3.3. Model Fit

The parallel piecewise LGCM and RI-CLPM were estimated using maximum likelihood with robust standard errors (MLR), and missing data were handled with full-information maximum likelihood (FIML). In both models, sex was included as a covariate to control for between-person sex differences in usual level. Both models showed acceptable fit (Table 3). Although CFI and TLI values did not reach the conventional threshold of 0.95, they exceeded 0.90, a criterion generally considered acceptable for complex longitudinal models with categorical or highly skewed outcomes (Hu & Bentler, 1999; Marsh et al., 2004). Given the six-wave parallel-process structure combined with the non-normal distribution of suicidal ideation (57–59% of participants at the lowest scale value across waves), this fit level is expected rather than a sign of misspecification.

In both models, Mplus returned a warning indicating that the latent covariance matrix was not positive definite; this reflects the intentional specification of fixing the variance of the LGCM step factor and the RI-CLPM COVID shift at zero to model a common mean-level change at the onset of the pandemic, and does not affect model identification or the interpretation of the main estimates. This constraint is theoretically motivated: the step factor represents an abrupt population-level shift shared by all individuals rather than an individually varying effect, and the warning is a technical byproduct of this specification rather than evidence of a problem with the model.

In the LGCM, an additional Heywood case occurred for the residual variance of the pre-COVID slope of suicidal ideation; the residual variance was therefore fixed at zero, as noted in Table 4. The unconstrained estimate was a small negative value (−0.007, SE = 0.011, *p* = 0.523) whose 95% confidence interval clearly included zero, indicating that the true population variance lies close to zero rather than being negative (F. Chen et al., 2001; Kolenikov & Bollen, 2012). Consistent with this interpretation, examination of the raw data revealed a strong floor effect at Waves 1–3: 37.6% of participants reported identical values across all three pre-COVID waves, and 52.9% showed no change between Waves 1 and 3. Such distributional features severely limit the individual variability that a pre-COVID slope factor can capture. Fixing the residual variance at zero is the standard treatment for such cases and does not affect the fixed-effect estimates, which are the parameters of primary substantive interest.

As a sensitivity check for the choice of Wave 3 as the pandemic breakpoint, a single-slope parallel-process LGCM assuming constant change across the six-year period without any breakpoint or step factor was estimated and compared with the primary piecewise model. The single-slope model showed poor fit (CFI = 0.824, TLI = 0.819, RMSEA = 0.053, SRMR = 0.070, AIC = 145,972.74, BIC = 146,207.12), with both CFI and TLI falling clearly below the acceptable threshold of 0.90 (Table 3). In contrast, the primary piecewise model showed acceptable fit on all indices (CFI = 0.929, TLI = 0.917, RMSEA = 0.036, SRMR = 0.035, AIC = 145,230.43, BIC = 145,576.13), yielding substantial differences in AIC (ΔAIC = 742.32) and BIC (ΔBIC = 630.99) in favor of the primary model (Supplementary Table S1). Furthermore, in the single-slope model, the slope-to-slope path from sleep quality to suicidal ideation was not significant (*B* = 0.077, *p* = 0.270), whereas in the primary model this path was significant at the pre-COVID slope but not at the post-COVID slope. This pattern indicates that a single-slope specification obscures the differential pre- and post-pandemic mediation dynamics identified in the primary analysis, providing empirical support for the specification of Wave 3 as the pandemic breakpoint.

**Table 3:** Model fit indices for the parallel piecewise LGCM and RI-CLPM.

Fit Index	LGCM	LGCM	RI-CLPM
	(parallel piecewise)	(single slope)	(with COVID shift)
χ^2^(*df*), *p*	633.47(148), *p* < 0.001	1365.35(167), *p* < 0.001	653.61(144), *p* < 0.001
CFI	0.929	0.824	0.925
TLI	0.917	0.819	0.911
RMSEA [90% CI]	0.036 [0.033, 0.038]	0.053 [0.050, 0.055]	0.037 [0.034, 0.040]
SRMR	0.035	0.070	0.043
AIC	145,230.43	145,972.74	145,260.17
BIC	145,576.13	146,207.12	145,629.32

Note. *N* = 2590. The parallel piecewise LGCM and RI-CLPM were estimated in Mplus 6.12 (Muthén & Muthén, 2010), and the single-slope LGCM was estimated in Mplus 8 (Muthén & Muthén, 2017); all models used maximum likelihood with robust standard errors (MLR) and full-information maximum likelihood (FIML) for missing data (Enders, 2022). Model fit was evaluated against the criteria recommended by Hu and Bentler (1999) (CFI/TLI ≥ 0.95, RMSEA ≤ 0.06, SRMR ≤ 0.08), with CFI/TLI ≥ 0.90 and RMSEA ≤ 0.08 considered acceptable for complex longitudinal models. LGCM = parallel piecewise latent growth curve model (Bollen & Curran, 2005) with the intercept centered at Wave 3 (2020) and a COVID-19 mean step at the latent level; sex was included as a covariate on the three intercept factors (SDep, SQ, SI) to control for stable between-person sex differences in usual level. The single-slope LGCM was estimated as a sensitivity analysis assuming constant change across the six-year period without any breakpoint or step factor; it is presented not as an alternative model but to demonstrate the empirical necessity of a breakpoint at Wave 3. RI-CLPM (Hamaker et al., 2015) = random-intercept cross-lagged panel model with a COVID-19 mean shift implemented at the observed-intercept level; sex was included as a covariate on the three random intercepts. CFI = comparative fit index; TLI = Tucker–Lewis index; RMSEA = root mean square error of approximation; SRMR = standardized root mean square residual; AIC = Akaike information criterion; BIC = Bayesian information criterion. Both models returned an Mplus warning indicating that the latent covariance matrix was not positive definite; this is by design, reflecting the constraint that the variance of the COVID-step factor (LGCM) and the COVID shift (RI-CLPM) was fixed at zero to model a common mean-level shift rather than an individually varying effect. In the LGCM, the residual variance of the SI pre-COVID slope was additionally fixed at zero to address a Heywood case (see Table 4; Kolenikov & Bollen, 2012).

**Table 4:** Latent growth factor means and variances in the parallel piecewise latent growth curve model, controlling for sex.

Construct	Growth Factor	Mean *B* ^a^	*SE*	*p*	Variance	*SE*	*P*
SDep	Intercept	33.233 ***	0.273	<0.001	28.104 ***	1.437	<0.001
	Pre-COVID slope	1.469 ***	0.156	<0.001	4.896 ***	0.605	<0.001
	COVID step	−0.734 **	0.251	0.003	0.000 fixed	—	—
	Post-COVID slope	−0.155 **	0.053	0.004	2.437 ***	0.251	<0.001
SQ	Intercept	3.826 ***	0.074	<0.001	0.129 ***	0.012	<0.001
	Pre-COVID slope	0.016	0.018	0.377	0.033 ***	0.006	<0.001
	COVID step	0.004	0.026	0.873	0.000 fixed	—	—
	Post-COVID slope	−0.047 ***	0.006	<0.001	0.020 ***	0.003	<0.001
SI	Intercept	1.228 ***	0.154	<0.001	0.142 ***	0.012	<0.001
	Pre-COVID slope	−0.104 ***	0.021	<0.001	0.000 fixed ^b^	—	—
	COVID step	0.043	0.030	0.151	0.000 fixed	—	—
	Post-COVID slope	0.027 ***	0.007	<0.001	0.012 ***	0.003	<0.001

Note. *N* = 2590. *B* = unstandardized estimate; SE = robust standard error. Sex was included as a covariate on the three intercept factors (SDep, SQ, SI) to control for stable between-person sex differences in usual level; it was not included on slope or step factors, consistent with random-intercept cross-lagged panel model evidence that sex effects operate at the level of stable individual differences (see Section 3.5). Sex was coded 0 (male) and 1 (female). The intercept was centered at Wave 3 (2020). The pre-COVID slope captured change across Waves 1–3 (loadings −2, −1, 0, 0, 0, 0); the post-COVID slope captured change across Waves 3–6 (loadings 0, 0, 0, 1, 2, 3); the COVID step captured the immediate mean-level shift at Wave 3 (loadings 0, 0, 1, 1, 1, 1) with its variance fixed at 0 to represent a common shift. SDep = smartphone dependence (sum score); SQ = weekday sleep quality (1 = very poorly, 4 = very well); SI = suicidal ideation (1 = strongly disagree, 4 = strongly agree). ^a^ For the intercept factors (SDep, SQ, SI), Mean B represents the regression intercept when covariates are at their reference values (male reference for sex, and predecessor growth factors at 0 where applicable). For pre- and post-COVID slope factors of SQ and SI, the reported Mean B likewise represents a regression intercept because these factors are regressed on the corresponding slope of SDep or SQ (see Table 5 for structural paths). ^b^ The residual variance of the SI pre-COVID slope was fixed at 0 to address a Heywood case in the unconstrained model (unconstrained estimate = −0.007, *SE* = 0.011, *p* = 0.523), reflecting the limited individual variability in pre-COVID change in SI associated with the floor effect at Waves 1–3 (see Section 3.3; F. Chen et al., 2001; Kolenikov & Bollen, 2012). ** p < 0.01; *** p < 0.001.

**Table 5:** Direct, indirect, and total effects in the parallel piecewise latent growth curve model, controlling for sex.

Level	Effect/Path	*B*	*SE*	β	*p*
Intercept	SDep → SQ	−0.021 ***	0.002	−0.298 ***	<0.001
	SQ → SI	−0.229 ***	0.035	−0.202 ***	<0.001
	SDep → SI (direct)	0.028 ***	0.002	0.347 ***	<0.001
	Indirect: SDep → SQ → SI	0.005 ***	0.001	—	<0.001
	Total effect	0.033 ***	0.002	—	<0.001
Pre-COVID slope	SDep → SQ	−0.021 ***	0.005	−0.247 ***	<0.001
	SQ → SI	−0.384 ***	0.097	−0.551 ***	<0.001
	SDep → SI (direct)	0.042 ***	0.007	0.709 ***	<0.001
	Indirect: SDep → SQ → SI	0.008 **	0.002	—	0.001
	Total effect	0.050 ***	0.007	—	<0.001
Post-COVID slope	SDep → SQ	−0.028 ***	0.005	−0.294 ***	<0.001
	SQ → SI	0.036	0.062	0.040	0.558
	SDep → SI (direct)	0.051 ***	0.006	0.597 ***	<0.001
	Indirect: SDep → SQ → SI	−0.001	0.002	—	0.570
	Total effect	0.050 ***	0.005	—	<0.001

Note. *N* = 2590. *B* = unstandardized estimate; SE = robust standard error; β = standardized (STDYX) estimate, reported only for individual regression paths. Sex was included as a covariate on the three intercept factors (SDep, SQ, SI) to control for stable between-person sex differences in usual level; it was not included on slope or step factors. Indirect effects (SDep → SQ → SI) and total effects (direct + indirect) were computed in the Mplus MODEL CONSTRAINT block. Effects are reported at three growth-factor levels: the intercept (between-person typical level), the pre-COVID slope (change across Waves 1–3), and the post-COVID slope (change across Waves 3–6). SDep = smartphone dependence; SQ = weekday sleep quality; SI = suicidal ideation. ** *p* < 0.01; *** *p* < 0.001.

### 3.4. Parallel Piecewise LGCM

The means and variances of the latent growth factors representing the six-year developmental trajectories of the three variables are presented in Table 4, and the mediation paths among slopes, direct and indirect effects, and total effects are presented in Table 5. In the LGCM, sex was included as a covariate on the three intercept factors to control for between-person sex differences in usual level.

The three variables followed different developmental trajectories. Smartphone dependence increased by approximately 1.47 points per year during the pre-COVID period (Waves 1–3; pre-COVID slope *B* = 1.47, *SE* = 0.16, *p* < 0.001), then shifted downward by approximately 0.73 points below the level predicted by the pre-COVID trend at the onset of the pandemic (Wave 3, 2020; step factor B = −0.73, *SE* = 0.25, *p* = 0.003), and subsequently declined during the post-COVID period (Waves 3–6; post-COVID slope *B* = −0.16, *SE* = 0.05, *p* = 0.004). For sleep quality, neither the pre-COVID change trend (*p* = 0.377) nor the COVID-related step change (*p* = 0.873) was significant; however, sleep quality worsened by approximately 0.05 points per year during the post-COVID period (post-COVID *B* = −0.05, *SE* = 0.01, *p* < 0.001). Suicidal ideation showed a decreasing trend during the pre-COVID period (pre-COVID *B* = −0.10, *SE* = 0.02, *p* < 0.001), with no immediate shift at the onset of the pandemic (*p* = 0.151), followed by a gradual increase during the post-COVID period (post-COVID *B* = 0.03, *SE* = 0.01, *p* < 0.001). With the exception of the pre-COVID slope of suicidal ideation, the variances of the intercept and slope factors of all three variables were statistically significant (*p* < 0.001), indicating individual differences in developmental trajectories and meeting the prerequisite for subsequent mediation analyses of the associations among slopes. However, a Heywood case occurred for the residual variance of the pre-COVID slope of suicidal ideation, which was estimated as a negative value (unconstrained estimate = −0.007, *SE* = 0.011, *p* = 0.523), and was therefore fixed at zero (see the note to Table 4; F. Chen et al., 2001; Kolenikov & Bollen, 2012); as described in Section 3.3, this near-zero residual variance more plausibly reflects the limited individual variability in pre-COVID change in suicidal ideation associated with the floor effect at Waves 1–3 than a complete explanation of this change by smartphone dependence and sleep quality.

The mediation results showed a distinct asymmetry across the pre- and post-COVID periods. The first mediation stage, the effect of smartphone dependence on sleep quality, was negative and significant at all three growth-factor levels—the intercept, the pre-COVID slope, and the post-COVID slope (β = −0.30 to −0.25, all *p* < 0.001; Table 5). Adolescents with higher usual levels of smartphone dependence had poorer usual sleep quality, and those whose smartphone dependence increased more (or decreased less) during both the pre- and post-COVID periods also experienced greater deterioration in sleep quality. The second mediation stage, the effect of sleep quality on suicidal ideation, was significant at the intercept level (β = −0.20, *p* < 0.001) and at the pre-COVID slope level (β = −0.55, *p* < 0.001), but not at the post-COVID slope level (β = 0.04, *p* = 0.558). The direct effect of smartphone dependence on suicidal ideation was significantly positive at all three levels (β = 0.35 to 0.71, all *p* < 0.001; Table 5).

This pattern was directly reflected in the indirect-effect estimates. The indirect effect from smartphone dependence through sleep quality to suicidal ideation was significant at the intercept level (*B* = 0.005, SE = 0.001, *p* < 0.001) and at the pre-COVID slope level (*B* = 0.008, SE = 0.002, *p* = 0.001), but not at the post-COVID slope level (*B* = −0.001, SE = 0.002, *p* = 0.570). The indirect association through sleep quality was observed for between-person differences in usual levels and in pre-COVID change, but not for post-COVID change, for which the path from sleep quality to suicidal ideation was not significant. However, the total effect of smartphone dependence on suicidal ideation remained significantly positive at all three levels (*B* = 0.033 to 0.050, all *p* < 0.001), indicating that the overall association between smartphone dependence and suicidal ideation was similar across the pre- and post-COVID periods.

Wald tests indicated that the associations involving sleep quality differed between the pre- and post-COVID slopes (Table 6). The joint test of equality for the a and b paths was significant, Wald χ^2^(2) = 11.63, *p* = 0.003. In the individual comparisons, the a path from smartphone dependence to sleep quality did not differ significantly between periods (difference = 0.007, SE = 0.007, *p* = 0.336), whereas the b path from sleep quality to suicidal ideation did (difference = −0.420, SE = 0.123, *p* = 0.001). The indirect effect was also significantly larger during the pre-COVID period than during the post-COVID period (difference = 0.009, SE = 0.003, *p* = 0.004). By contrast, neither the direct effect (*p* = 0.314) nor the total effect (*p* = 0.993) differed significantly. These findings support a formal statistical difference in the mediating association across periods, although as an observational analysis they cannot establish that the pandemic itself produced this change.

To evaluate whether the primary model’s treatment of sex as an intercept-only covariate adequately captured sex-related patterns, the parallel piecewise LGCM was re-estimated separately for male and female subsamples as a robustness check. Both subsample models showed acceptable fit (males: CFI = 0.916, TLI = 0.905, RMSEA = 0.036, SRMR = 0.041; females: CFI = 0.939, TLI = 0.932, RMSEA = 0.038, SRMR = 0.041). Sex differences in the mediation paths (a, b, c’) and indirect effects were largely non-significant, supporting the primary model’s specification. Four coefficients differed by sex. The pre-COVID first-stage path (SDep → SQ) was significant only for female adolescents (z = 2.23, *p* = 0.026), and the post-COVID mean slope of smartphone dependence declined more steeply among female adolescents than among male adolescents (z = 2.75, *p* = 0.006). For suicidal ideation, the pre-COVID mean slope declined more steeply among female adolescents (z = 2.46, *p* = 0.014), and a significant upward shift at the onset of the pandemic was observed only among female adolescents (z = −3.62, *p* < 0.001). Detailed results are presented in Supplementary Table S3.

### 3.5. Random-Intercept Cross-Lagged Panel Model

The RI-CLPM examines how within-person deviations at a given time point predict other variables at the subsequent time point while controlling for stable between-person differences. The variances and covariances of the random intercepts, within-person autoregressive and cross-lagged paths, and estimates of the COVID-19 mean-level shift are presented in Table 7.

The COVID-19 mean-level shift differed across variables. Compared with the two pre-COVID waves (Waves 1–2), the post-COVID mean (Waves 3–6) was approximately 1.22 points higher for smartphone dependence (*B* = 1.22, *p* < 0.001) and approximately 0.07 points lower for sleep quality (*B* = −0.07, *p* < 0.001). No statistically significant shift in the mean level was observed for suicidal ideation (*p* = 0.400).

At the between-person stable level, significant random-intercept variance was observed for all three variables (all *p* < 0.001), indicating individual differences in usual levels among adolescents. Correlations among the random intercepts were statistically significant for all three pairs and showed theoretically consistent directions. Adolescents with higher usual smartphone dependence had poorer usual sleep quality (*r* = −0.31) and higher usual suicidal ideation (*r* = 0.32), and adolescents with better usual sleep quality had lower usual suicidal ideation (*r* = −0.29, all *p* < 0.001). When sex was included as a covariate, female adolescents showed higher usual levels of smartphone dependence (β = 0.07, *p* = 0.004) and suicidal ideation (β = 0.18, *p* < 0.001) than male adolescents, whereas no significant sex difference was observed for usual sleep quality (*p* = 0.705).

At the within-person level, the autoregressive paths for all three variables were positive and significant (all *p* < 0.001), indicating that individuals’ deviations from their usual levels at a given time point partly persisted into the next time point. Among the six cross-lagged paths, only the path from smartphone dependence to subsequent suicidal ideation was significant (β = 0.05, *p* < 0.001). When adolescents’ smartphone dependence at a specific time point was higher than their usual level, their suicidal ideation at the following time point also tended to be higher than their usual level. However, the paths from smartphone dependence to subsequent sleep quality and from sleep quality to subsequent suicidal ideation were not significant (all *p* > 0.17), and the within-person time-lagged mediating effect through sleep quality was therefore not supported. The paths from suicidal ideation and sleep quality to subsequent smartphone dependence were also not significant (all *p* > 0.20). Within-wave correlations among the within-person deviations were generally in the expected directions, with somewhat stronger associations during the two pre-COVID waves.

## 4. Discussion

This study used six-year longitudinal data from the middle school first-grade cohort of KCYPS 2018 to examine the developmental trajectories of smartphone dependence, sleep quality, and suicidal ideation among adolescents and the changes in these trajectories before and after the onset of the COVID-19 pandemic. In addition, the longitudinal relationships among the three variables were tested using a parallel piecewise latent growth curve model (LGCM) and a random-intercept cross-lagged panel model (RI-CLPM). The main findings are as follows. First, the three variables followed distinct developmental trajectories, with pronounced changes observed around the onset of the pandemic. Second, the pathway from smartphone dependence to suicidal ideation mediated by sleep quality was significant only at the level of the intercept and the pre-COVID developmental trajectory, but not during the post-COVID period. Because these growth factors capture differences in usual levels and rates of change between adolescents, this finding describes between-person associations. Third, at the within-person time-varying level with annual intervals, only the direct pathway from smartphone dependence to suicidal ideation was significant, and no mediating role of sleep quality was observed. The contribution of this study lies in suggesting that the ways in which smartphone dependence and sleep quality relate to suicidal ideation differ depending on the level of analysis (Hamaker et al., 2015; Rohrer & Murayama, 2023). In a setting where smartphone overdependence is highly prevalent among adolescents (Ministry of Science and ICT, 2026), self-perceived smartphone dependence co-occurred with poorer sleep quality and higher suicidal ideation between adolescents, whereas a year-to-year chain from dependence through sleep to suicidal ideation was not observed within adolescents.

The three variables followed different developmental trajectories. Smartphone dependence increased during the pre-COVID middle school period, consistent with the high prevalence of problematic smartphone use among children and young people (Sohn et al., 2019). However, at the onset of the pandemic, contrary to expectations, smartphone dependence shifted to a level lower than that predicted by the pre-COVID trend. Because the step factor represents a deviation from the extrapolated pre-COVID linear trend, this result does not indicate an absolute decrease; the observed means remained in the low 32-point range from Wave 2 through Wave 6 (Section 3.1), indicating a plateau with slight decline rather than a marked decrease in mid-adolescence. This pattern corresponds to national screening data in which the number of first-year high school students at risk of Internet and smartphone overdependence decreased in 2021, the year in which the present cohort entered high school, whereas it increased among fourth-grade elementary school students (Ministry of Gender Equality and Family, 2021). The plateau may also reflect what the scale measures. Problematic smartphone use is defined by impaired control and interference with daily functioning rather than by time spent on the device (Billieux, 2012; Panova & Carbonell, 2018). When smartphones became necessary for remote classes and school communication, adolescents may have been less likely to perceive their use as interfering with daily life. This interpretation could not be tested with the present data, which did not include measures of usage time or purpose. However, recent research suggests that adolescents’ smartphone use should not be treated as a single quantity, because the impact on mental health differs depending on the purpose of use (passive consumption vs. active communication) (S. Kim et al., 2020); it should therefore be noted that a decrease in the total level of smartphone dependence does not necessarily indicate a reduction in risk. Sleep quality showed no significant change during the pre-COVID period but worsened moderately after the pandemic, which may reflect the transition to high school and the academic demands of preparing for college entrance examinations. In a national survey conducted in 2020, Korean adolescents slept 6.3 h on average, and only 30.8% were satisfied with their sleep (D. H. Kim et al., 2022). Sleep satisfaction in national data was nevertheless higher in 2020 than in 2019 (J. Lee et al., 2022), suggesting that the pandemic itself may not account for the decline observed in the present cohort. Suicidal ideation decreased during the pre-COVID period and then increased after the pandemic. The step at the pandemic onset was not significant, which is consistent with national data showing lower suicidal ideation among students in 2020 than in 2019 (J. Lee et al., 2022). The subsequent gradual increase resembles the pattern reported in an interrupted time-series analysis, in which suicidal ideation decreased in the first year of the pandemic and increased again in the second and third years (Y. Kim et al., 2025). Because these changes coincided with age-related development in a single cohort, however, they cannot be attributed to the pandemic.

At the developmental trajectory level, mediation through sleep quality was significant. In the parallel piecewise LGCM, the indirect pathway from smartphone dependence to suicidal ideation through sleep quality was significant at both the intercept level and the pre-COVID slope level. These findings are consistent with prior research linking greater smartphone use to poorer self-reported sleep quality (Chu et al., 2023), prospective studies of adolescents’ device use, sleep, and mental health (Dibben et al., 2023), and with meta-analytic evidence identifying sleep problems as a longitudinal risk factor for suicidal thoughts and behaviors (J.-W. Liu et al., 2019; R. T. Liu et al., 2020). However, because the residual variance of the pre-COVID slope of suicidal ideation was fixed at zero to address a Heywood case (Kolenikov & Bollen, 2012) and pre-COVID change in suicidal ideation showed limited individual variability owing to floor effects, the slope-level mediation during this period should be interpreted with caution. During the post-COVID period, however, the path from sleep quality to suicidal ideation was not significant, and the indirect effect disappeared. The Wald tests confirmed that the path from sleep quality to suicidal ideation and the indirect effect differed between the two periods, whereas the path from smartphone dependence to sleep quality did not (Table 6). Rather than indicating that the mediating role of sleep quality has vanished entirely, this pattern may reflect developmental conditions in high school. When short sleep becomes common among most students because of academic schedules, differences in perceived sleep quality between adolescents may carry less information about psychological distress. Factors not assessed in the present study, such as social isolation, family conflict, and academic uncertainty, may also have contributed to changes in suicidal ideation during this period. These explanations are post hoc and require testing in studies that measure these factors directly.

A different pattern emerged at the within-person, annually spaced time-varying level. In the RI-CLPM, the random intercepts, which represent between-person stable levels, were significantly related for all three variables, consistent with the intercept-level results from the LGCM. However, at the within-person cross-lagged level, only the path from smartphone dependence to subsequent suicidal ideation was significant. The RI-CLPM allows the separation of between-person stable levels from within-person time-varying change (Hamaker et al., 2015; Rohrer & Murayama, 2023); the finding that the three variables were related at the level of the random intercepts but not at the level of within-person cross-lagged paths indicates that stable between-person associations and within-person lagged associations may reflect different psychological processes. The mediation observed at the developmental trajectory level in the LGCM does not necessarily translate into within-person lagged associations in the RI-CLPM. A similar distinction has been reported for social media use: in an eight-year study, increases in an adolescent’s time spent on social media were not associated with subsequent changes in that adolescent’s depression or anxiety (Coyne et al., 2020). In the present study, neither the path from smartphone dependence to sleep quality nor the path from sleep quality to smartphone dependence was significant at the within-person level. This differs from a recent three-wave analysis of the same panel, in which poorer sleep quality predicted later smartphone overdependence (Oh & Heo, 2026); because that analysis did not separate stable between-person differences from within-person change, its lagged paths may partly reflect stable individual differences (Hamaker et al., 2015). The present findings therefore suggest that smartphone dependence and poor sleep quality co-occur among the same adolescents, but they do not establish which precedes the other from one year to the next.

The absence of within-person mediation should not be taken as evidence that sleep is unrelated to suicidal ideation within individuals. Prospective studies of sleep and suicidal thoughts and behaviors have mostly used follow-up intervals of a month or longer, and few have assessed short-term risk (R. T. Liu et al., 2020). In contrast, intensive monitoring studies have found that poorer sleep predicts next-day suicidal ideation among adolescents and young adults at high risk for suicide (Hamilton et al., 2023) and that negative social media experiences, but not screen time, are associated with daily suicidal ideation among adolescents (Hamilton et al., 2024). If such processes unfold over days, annual assessments may be too widely spaced to detect them. The present data contain no short-interval measurements, however, so this possibility remains untested (Hamaker & Wichers, 2017). The autoregressive effects were strongest for smartphone dependence, allowing the next time point to be predicted from the current value, but were smaller for suicidal ideation, indicating that suicidal ideation may fluctuate over time and may therefore require repeated assessment; intensive monitoring studies have likewise shown that suicidal ideation varies from day to day within adolescents and young adults (Hamilton et al., 2023).

With respect to sex differences, similar patterns were observed in both models. Female adolescents showed higher usual levels of smartphone dependence and suicidal ideation than male adolescents, whereas no significant sex difference was observed for sleep quality. In the separate models for male and female adolescents, the pre-COVID path from smartphone dependence to sleep quality was significant only among female adolescents, and smartphone dependence declined more steeply after the pandemic onset among female adolescents. Among female adolescents, suicidal ideation also decreased more steeply before the pandemic and showed a significant upward shift at its onset, whereas no such shift was observed among male adolescents (Table S3). This finding may reflect that female adolescents use smartphones as a means of maintaining peer relationships and expressing emotions and may therefore be more affected by negative digital experiences such as social comparison, relational conflict, and cyberbullying (John et al., 2018; Nesi et al., 2018). Recent neuroscientific research has suggested that specific periods of adolescence represent developmental windows of heightened vulnerability to social stimuli from social media (Orben & Blakemore, 2023). In a large UK sample, these windows occurred earlier in girls (ages 11–13) than in boys (ages 14–15) (Orben et al., 2022), which overlaps with the beginning of the present observation period and is consistent with the higher levels of smartphone dependence and suicidal ideation observed among girls in the present study. The absence of sex differences in sleep quality suggests that sleep problems among Korean adolescents may be more strongly related to shared environmental factors such as academic burden, school start time, and life rhythm than to sex.

The present findings have several implications for prevention, although associations observed in panel data cannot by themselves establish whether screening or intervention would be beneficial. First, the between-person findings bear on whom to attend to rather than on how risk is transmitted. Adolescents who report high smartphone dependence together with poor sleep quality may warrant attention as a group with elevated suicidal ideation. In South Korea, this does not require a new population screening program. Screening for Internet and smartphone overdependence has been conducted annually since 2009 for students in the fourth grade of elementary school and the first year of middle and high school, with counseling and treatment services linked to students at risk (Ministry of Gender Equality and Family, 2021), and a separate school-based screening for emotional and behavioral problems is administered in the same grades (I. T. Kim et al., 2017). The present findings suggest that interpreting these two screening results together may be more informative than treating them separately. Caution is nonetheless warranted. The US Preventive Services Task Force concluded that the evidence is insufficient to assess the balance of benefits and harms of screening children and adolescents for suicide risk (US Preventive Services Task Force, 2022), and in a Korean survey of parents whose children had been identified as at risk through school screening, concern about stigma was the most frequently cited area for improvement (I. T. Kim et al., 2017). Positive screening results should therefore lead to further assessment and support rather than to labeling. Because within-person increases in smartphone dependence preceded higher suicidal ideation in the following year, attention from teachers and school counselors between screening years may complement screening conducted only at school transition points.

Second, regarding interventions, the present findings do not indicate that improving sleep in a given year would reduce suicidal ideation in the following year, because no within-person pathway through sleep quality was observed. Poor sleep nevertheless warrants attention in its own right, and school-based cognitive behavioral therapy for insomnia has been shown to improve self-reported sleep quality and duration among adolescents with insomnia symptoms (Ewart et al., 2025). Sleep-focused interventions should extend beyond one-time educational sessions to include long-term changes in living environments, such as the establishment of regular sleep habits, management of nighttime smartphone use, and adjustment of academic schedules (van den Eijnden et al., 2021). Evidence for interventions targeting problematic smartphone use in adolescence remains limited, although brief school-based programs are being developed; in a preliminary trial, a five-session preventive program based on the metacognitive model reduced daily phone use and risky smartphone behaviors among high school students (Donati et al., 2025). Restricting phone use at school, which many countries have introduced, was not associated with better mental wellbeing, sleep, or lower overall phone use in a study of English schools (Goodyear et al., 2025), suggesting that interventions may need to address how adolescents use their phones outside school rather than access alone. In addition, interventions targeting smartphone dependence should evaluate not only the total amount of use but also qualitative aspects such as the purposes of gaming and social media use, social comparison, cyberbullying experiences, and fear of missing out (FOMO) (Elhai et al., 2016; John et al., 2018; S. Kim et al., 2020; Nesi et al., 2018). This recommendation is consistent with evidence that the experiences adolescents have on social media, rather than the time they spend on it, are associated with daily suicidal ideation (Hamilton et al., 2024).

Third, regarding preparedness for future pandemics, the present design cannot isolate the effects of the pandemic from age-related development, and the findings should not be read as estimates of pandemic effects. Two considerations nevertheless follow. Changes in self-reported dependence scores during periods of remote schooling may partly reflect changes in how necessary and interfering smartphone use is perceived to be, so monitoring during such periods may benefit from combining self-reported dependence with information on the purposes of use and daily functioning. In addition, because the association between sleep quality and suicidal ideation differed across periods, the indicators that are informative for identifying adolescents at elevated risk may themselves change with circumstances, which argues for periodically re-examining screening indicators rather than assuming that they remain stable.

This study has several limitations. First, because the analysis was based on a single cohort design, it is difficult to fully disentangle the effects of the COVID-19 pandemic from the developmental effects associated with age (e.g., the transition to high school and the burden of preparing for college entrance examinations). The step and post-COVID slope estimates assume that the pre-COVID linear trend would have continued; if developmental change naturally decelerated in mid-adolescence, as the plateau in smartphone dependence may suggest, part of what these estimates attribute to the post-onset period reflects age rather than period. Multi-cohort designs that compare adolescents of the same age before and after the pandemic are needed to separate these effects. Second, sleep quality and suicidal ideation were each assessed with a single self-report item, limiting the aspects of these constructs that could be examined and precluding estimates of internal consistency. Potential measurement error may affect the estimated indirect effects (Millner et al., 2015; VanderWeele et al., 2012). These effects should therefore be interpreted as associations among the available measures, rather than evidence of an established mediating mechanism. Third, all variables were self-reported, and adolescents’ perceptions of their own smartphone use may be colored by their mood; shared method variance may therefore have inflated the associations among the three variables. Future studies could combine self-reports with observable behavioral data; in a school-based sample of adolescents, combining self-reported and passively sensed smartphone data improved the prediction of suicidal ideation compared with self-reported data alone (Kadirvelu et al., 2026). Fourth, the smartphone dependence scale did not distinguish the purposes of use, such as social media, gaming, or information seeking. Because negative social media experiences such as social comparison have been associated with suicidal ideation (Hamilton et al., 2024), future research should measure what adolescents do on their phones as well as how dependent they feel. Fifth, psychosocial factors that may have influenced suicidal ideation following COVID-19—such as social isolation, family conflict, and changes in peer relationships—were not directly measured, and depressive symptoms were not included in the models. Sixth, in the RI-CLPM, time-invariance constraints were imposed on the autoregressive and cross-lagged paths for parsimony and estimation stability (Mulder & Hamaker, 2021), which limits the ability to test changes in the within-person relational structure before and after COVID-19. Seventh, because the present study examined within-person variation at annual intervals, associations between sleep quality and suicidal ideation within sub-annual periods—such as differences in life rhythm between vacations and school semesters—could not be examined (Hamaker & Wichers, 2017). Accordingly, the present findings cannot distinguish short-term from long-term processes. Eighth, FIML assumes that data are missing at random; although retained and non-retained participants did not differ on the outcome variables at baseline, attrition related to unobserved changes in these variables cannot be excluded. Future research should include comparison cohorts, multidimensional measures, psychosocial variables, and time-varying and multi-group models to further test the present findings.

In conclusion, across six years from early to late adolescence, self-perceived smartphone dependence, poorer sleep quality, and suicidal ideation co-occurred among the same adolescents, and the association between smartphone dependence and suicidal ideation was consistent with an indirect pathway through sleep quality at the between-person level. Within adolescents, however, a higher-than-usual level of smartphone dependence was followed by higher suicidal ideation in the next year without a corresponding pathway through sleep quality. These findings suggest that, at present, the value of these indicators lies mainly in identifying which adolescents may need attention within existing school-based screening systems, rather than in specifying a mechanism to target through intervention. Future research should examine how and when risk is transmitted by measuring the purposes and experiences of smartphone use, combining self-reports with observable behavioral data, assessing sleep and suicidal ideation at shorter intervals, and using multi-cohort designs to separate developmental and period effects.

## Figures and Tables

**Table 1:** Descriptive statistics and Pearson correlations for study variables across six annual waves (*N* = 2590).

Variable		1	2	3	4	5	6	7	8	9	10	11	12	13	14	15	16	17	18
SDep T1	1.	1																	
SDep T2	2.	0.42 ***	1																
SDep T3	3.	0.35 ***	0.47 ***	1															
SDep T4	4.	0.27 ***	0.41 ***	0.47 ***	1														
SDep T5	5.	0.23 ***	0.35 ***	0.41 ***	0.47 ***	1													
SDep T6	6.	0.21 ***	0.32 ***	0.37 ***	0.42 ***	0.58 ***	1												
SQ T1	7.	−0.15 ***	−0.14 ***	−0.10 ***	−0.10 ***	−0.06 **	−0.06 **	1											
SQ T2	8.	−0.06 **	−0.17 ***	−0.08 ***	−0.11 ***	−0.10 ***	−0.09 ***	0.27 ***	1										
SQ T3	9.	−0.09 ***	−0.11 ***	−0.13 ***	−0.10 ***	−0.10 ***	−0.07 ***	0.23 ***	0.27 ***	1									
SQ T4	10.	−0.07 ***	−0.07 ***	−0.04 *	−0.12 ***	−0.11 ***	−0.09 ***	0.16 ***	0.21 ***	0.35 ***	1								
SQ T5	11.	−0.02	−0.04 *	−0.06 **	−0.06 **	−0.05 **	−0.07 ***	0.15 ***	0.17 ***	0.27 ***	0.28 ***	1							
SQ T6	12.	−0.02	−0.08 ***	−0.02	−0.10 ***	−0.13 ***	−0.15 ***	0.09 ***	0.13 ***	0.23 ***	0.26 ***	0.42 ***	1						
SI T1	13.	0.25 ***	0.15 ***	0.13 ***	0.09 ***	0.06 **	0.07 ***	−0.21 ***	−0.09 ***	−0.09 ***	−0.04 *	−0.04 *	−0.04	1					
SI T2	14.	0.10 ***	0.30 ***	0.13 ***	0.15 ***	0.08 ***	0.07 ***	−0.09 ***	−0.17 ***	−0.11 ***	−0.06 **	−0.05 *	−0.07 ***	0.21 ***	1				
SI T3	15.	0.09 ***	0.17 ***	0.20 ***	0.13 ***	0.10 ***	0.12 ***	−0.06 **	−0.11 ***	−0.13 ***	−0.06 **	−0.05 *	−0.06 **	0.21 ***	0.30 ***	1			
SI T4	16.	0.06 **	0.16 ***	0.12 ***	0.21 ***	0.11 ***	0.08 ***	−0.06 **	−0.08 ***	−0.10 ***	−0.10 ***	−0.03	−0.00	0.13 ***	0.24 ***	0.31 ***	1		
SI T5	17.	−0.01	0.06 **	0.04 *	0.06 **	0.18 ***	0.09 ***	−0.00	−0.05 **	−0.06 **	−0.07 ***	−0.10 ***	−0.09 ***	0.10 ***	0.15 ***	0.24 ***	0.28 ***	1	
SI T6	18.	0.01	0.07 ***	0.07 ***	0.09 ***	0.18 ***	0.23 ***	−0.01	−0.04 *	−0.06 **	−0.02	0.01	−0.06 **	0.05 *	0.12 ***	0.17 ***	0.21 ***	0.27 ***	1
*M*		30.58	32.05	32.78	32.41	32.80	32.19	3.15	3.14	3.14	3.08	3.01	3.02	1.56	1.53	1.52	1.56	1.58	1.58
*SD*		7.32	7.06	7.40	6.94	6.58	6.40	0.67	0.63	0.63	0.64	0.62	0.70	0.75	0.71	0.69	0.67	0.69	0.67

Note. SDep = smartphone dependence (sum score); SQ = weekday sleep quality (1 = very poorly, 4 = very well); SI = suicidal ideation (1 = strongly disagree, 2 = somewhat disagree, 3 = somewhat agree, 4 = strongly agree); T1–T6 = annual assessments from 2018 to 2023, with T3 (2020) coinciding with the COVID-19 pandemic onset. Means, standard deviations, and correlations were estimated under full-information maximum likelihood (FIML). Correlations are based on Mplus model-estimated sample statistics. Variable numbers (1–18) correspond to row order. * *p* < 0.05. ** *p* < 0.01. *** *p* < 0.001.

**Table 2:** Longitudinal measurement invariance of the smartphone dependence scale across six annual waves.

Model	χ^2^	*df*	CFI	TLI	RMSEA	SRMR	ΔCFI	ΔRMSEA
Configural	14,032.35	3537	0.913	0.901	0.040	0.048	—	—
Metric (full)	—	—	0.859	0.843	0.050	0.068	−0.054	+0.010
Metric (partial) ^a^	13,798.16	3591	0.915	0.905	0.039	0.050	+0.002	−0.001
Scalar (partial) ^b^	14,410.48	3666	0.911	0.903	0.040	0.048	−0.004	+0.001

Note. *N* = 2590. Longitudinal measurement invariance was tested by sequentially imposing equality constraints across the six annual waves on a four-factor model of smartphone dependence (disturbance of adaptive functions, virtual life orientation, withdrawal, and tolerance). χ^2^ values are the mean- and variance-adjusted (scaled) test statistic estimated with WLSMV. ΔCFI and ΔRMSEA for the full and partial metric models are computed relative to the configural model; ΔCFI and ΔRMSEA for the partial scalar model are computed relative to the partial metric model. The full metric model was rejected (ΔCFI > 0.010) and is shown for comparison only; it was not carried forward. Model comparisons were evaluated using the criteria of F. F. Chen (2007). CFI = comparative fit index; TLI = Tucker–Lewis index; RMSEA = root mean square error of approximation; SRMR = standardized root mean square residual. ^a^ Factor loadings for items 5, 9, 10, 12, 13, and 15 were freed at Wave 4 only; all other loadings were constrained to equality across the six waves. ^b^ Item thresholds were constrained to equality across waves in addition to the partial loading pattern described in ^a^.

**Table 6:** Wald tests of mediation coefficient differences between pre- and post-COVID slopes in the parallel piecewise latent growth curve model.

Parameter	Difference (*B*)	SE	*p*
Individual paths			
SDep → SQ (a path)	0.007	0.007	0.336
SQ → SI (b path)	−0.420 **	0.123	0.001
SDep → SI (direct, c′)	−0.009	0.009	0.314
Mediation and total effects			
Indirect: SDep → SQ → SI (a × b)	0.009 **	0.003	0.004
Total effect (direct + indirect)	0.000	0.008	0.993

Note. *N* = 2590. Differences represent pre-COVID minus post-COVID unstandardized coefficients, computed in Mplus using MODEL CONSTRAINT within the primary parallel piecewise LGCM. Positive values indicate that the pre-COVID coefficient is larger than the post-COVID coefficient. The individual pre-COVID and post-COVID coefficients used to compute these differences are reported in Table 5. A joint Wald test evaluating the null hypothesis that both the a path (SDep → SQ) and the b path (SQ → SI) are equal across the two periods was significant, Wald χ^2^(2) = 11.63, *p* = 0.003, indicating that at least one of the two mediation-related paths differed between the periods. Sex was included as a covariate on the three intercept factors (SDep, SQ, SI), consistent with the primary model. *B* = unstandardized estimate; SE = robust standard error. SDep = smartphone dependence; SQ = weekday sleep quality; SI = suicidal ideation. a = first-stage path (SDep → SQ); b = second-stage path (SQ → SI); c′ = direct path (SDep → SI). ** *p* < 0.01.

**Table 7:** Random-intercept cross-lagged panel model results with COVID-19 shift.

Parameter	*B*	*SE*	β/*r*	*p*
***COVID shift (post-COVID mean vs. pre-COVID average)***				
kSDep (smartphone dependence shift)	1.218 ***	0.116	—	<0.001
kSQ (sleep quality shift)	−0.072 ***	0.011	—	<0.001
kSI (suicidal ideation shift)	0.010	0.012	—	0.400
***Between-person: Random-intercept parameters***				
Var (RI_SDep)	15.017 ***	0.835	—	<0.001
Var (RI_SQ)	0.076 ***	0.006	—	<0.001
Var (RI_SI)	0.073 ***	0.007	—	<0.001
Cov (RI_SDep, RI_SQ)	−0.328 ***	0.050	−0.307 ***	<0.001
Cov (RI_SDep, RI_SI)	0.337 ***	0.052	0.322 ***	<0.001
Cov (RI_SQ, RI_SI)	−0.021 ***	0.004	−0.286 ***	<0.001
RI_SDep ON sex (female = 1)	0.571 **	0.196	0.073 **	0.004
RI_SQ ON sex (female = 1)	0.006	0.016	0.011	0.705
RI_SI ON sex (female = 1)	0.099 ***	0.017	0.180 ***	<0.001
***Within-person autoregressive paths (time-invariant)***				
SDep → SDep	0.221 ***	0.016	0.231 ***	<0.001
SQ → SQ	0.155 ***	0.016	0.155 ***	<0.001
SI → SI	0.118 ***	0.016	0.121 ***	<0.001
***Within-person cross-lagged paths (time-invariant)***				
SQ → SDep	−0.121	0.124	−0.013	0.328
SI → SDep	0.139	0.112	0.017	0.217
SDep → SQ	0.000	0.001	0.005	0.706
SI → SQ	−0.014	0.011	−0.016	0.219
SDep → SI	0.005 ***	0.001	0.049 ***	<0.001
SQ → SI	−0.018	0.014	−0.017	0.177

Note. *N* = 2590. The RI-CLPM decomposes each observed score into a between-person random intercept (RI) and a time-specific within-person deviation (w). Autoregressive and cross-lagged paths were constrained to be equal across the five lagged transitions (Waves 1→2 through 5→6). The COVID shift was implemented at the observed-intercept level by constraining Wave 3–6 intercepts to be equal per variable; k parameters are derived in MODEL CONSTRAINT as the difference between the post-COVID intercept and the average of the two pre-COVID intercepts (Wave 1 and Wave 2). Sex was coded 0 (male) and 1 (female). *B* = unstandardized estimate; *SE* = robust standard error; β = standardized (STDYX) regression coefficient; *r* = standardized correlation. For autoregressive paths, the standardized solution shown is the mean of the five wave-specific standardized values (range varied within ±0.02). Random-intercept covariances are reported with unstandardized estimates (*B*) and standardized correlations (*r*). SDep = smartphone dependence; SQ = weekday sleep quality; SI = suicidal ideation. Within-wave residual correlations among the three latent within-person factors were significant and in the expected directions at most waves (SDep–SQ: *r* range −0.13 to 0.02; SDep–SI: *r* range 0.13 to 0.28; SQ–SI: r range −0.19 to −0.02), with somewhat stronger associations during the two pre-COVID waves. ** *p* < 0.01; *** *p* < 0.001.

## Data Availability

The datasets analyzed during the current study are publicly available from the Korean Children and Youth Panel Survey (KCYPS 2018), conducted by the National Youth Policy Institute of Korea. The data can be accessed from the National Youth Policy Institute Data Archive at https://www.nypi.re.kr/archive (accessed on 30 September 2026).

## Supplementary Materials

The following supporting information can be downloaded at https://www.mdpi.com/article/10.3390/bs16101828/s1, Table S1: Sensitivity analysis: comparison of the primary parallel piecewise LGCM with a single-slope LGCM; Table S2: Attrition analysis comparing participants retained at Wave 6 with those who dropped out on their Wave 1 characteristics; Table S3: Parallel piecewise LGCM estimates by sex subsample and z-tests of sex differences.

## Abbreviations

The following abbreviations are used in this manuscript:

KCYPS	Korean Children and Youth Panel Survey
LGCM	Latent Growth Curve Model
RI-CLPM	Random-Intercept Cross-Lagged Panel Model
SCL-90-R	Symptom Checklist-90-Revised
FIML	Full-Information Maximum Likelihood
MLR	Maximum Likelihood with Robust standard errors
CFI	Comparative Fit Index
TLI	Tucker–Lewis Index
RMSEA	Root Mean Square Error of Approximation
SRMR	Standardized Root Mean Square Residual
SDep	Smartphone Dependence
SQ	Weekday Sleep Quality
SI	Suicidal Ideation
AIC	Akaike Information Criterion
BIC	Bayesian Information Criterion
