# Supplementary material for: Smartphone Dependence, Sleep Quality, and Suicidal Ideation Across Adolescence: A Six-Year Longitudinal Study
Source: Behav Sci (Basel). 2026 Oct 6;16(10):1828. doi: 10.3390/bs16101828 (PMC13701268; doi:10.3390/bs16101828)
Supplement: Supplementary file 1 [file behavsci-16-01828-s001.zip › behavsci-4540870-supplementary.pdf]

Supplementary Material

Table S1. Sensitivity analysis: Comparison of the primary parallel piecewise LGCM with a single-slope LGCM.

Model	Free parameters	χ^2	*df*	CFI	TLI	RMSEA	SRMR	AIC	BIC
Primary: piecewise LGCM (Wave 3 breakpoint + step factor)	59	633.47	148	.929	.917	.036	.035	145,230.43	145,576.13
Sensitivity: single-slope LGCM (no breakpoint, no step)	40	1365.35	167	.824	.819	.053	.070	145,972.74	146,207.12
Difference (Primary − Sensitivity)	+19	—	—	+.105	+.098	−.017	−.035	−742.32	−630.99

Note. N = 2,590. Both models were estimated in Mplus using maximum likelihood with robust standard errors (MLR) and full-information maximum likelihood (FIML) for missing data. The primary model is a parallel piecewise LGCM with Wave 3 (2020) as the pandemic breakpoint, three growth factors per variable (intercept, pre-COVID slope, post-COVID slope), and a COVID step factor capturing the abrupt mean-level shift at the pandemic onset. The sensitivity model is a parallel single-slope LGCM assuming constant change across the six-year period without any breakpoint or step factor. Positive differences in CFI/TLI and negative differences in RMSEA/SRMR/AIC/BIC all favor the primary model. Chi-square values are the MLR-scaled test statistic. Chi-square and df are not comparable between MLR models with different scaling factors. CFI = comparative fit index; TLI = Tucker–Lewis index; RMSEA = root mean square error of approximation; SRMR = standardized root mean square residual; AIC = Akaike information criterion; BIC = Bayesian information criterion. Leading zeros for values less than 1 are omitted.

In the single-slope model, the slope-to-slope path from sleep quality to suicidal ideation was not significant (B = 0.077, p = .270), whereas in the primary model this path was significant at the pre-COVID slope (β = −.55, p < .001) but not at the post-COVID slope. This pattern indicates that a single-slope specification obscures the differential pre- and post-pandemic mediation dynamics identified in the primary analysis.

Table S2. Attrition analysis comparing participants retained at Wave 6 with those who dropped out on their Wave 1 characteristics.

Wave 1 variable	Retained (n = 2,224)	Dropped (n = 366)	*t*	*df*	*p*	Cohen's *d*
Smartphone dependence	30.63 (7.34)	30.31 (7.22)	0.78	484.22	.438	0.044
Sleep quality	3.14 (0.67)	3.19 (0.63)	−1.38	513.25	.169	−0.074
Suicidal ideation	1.57 (0.76)	1.52 (0.75)	1.17	495.99	.244	0.065
Sex (% girls)	46.6%	40.4%	4.61	1	.032	0.042

Note. N = 2,590. Retained participants were defined as those with valid data on at least one of the three main outcome variables (smartphone dependence, sleep quality, suicidal ideation) at Wave 6. Dropped participants were those with missing data on all three main outcome variables at Wave 6. Values in the Retained and Dropped columns for continuous variables are means (standard deviations). For the Sex row, values are the proportion of girls, and the chi-square test compared the sex distribution between retained and dropped groups (χ^2(1, N = 2,590) = 4.61, p = .032, Cramér's V = 0.042). Welch's t-tests were used for continuous variables to account for potentially unequal variances. Cohen's d values indicate small effect sizes (|d| < 0.10), suggesting that selective attrition on the main outcome variables was minimal. Leading zeros for values less than 1 are omitted.

Table S3. Parallel piecewise LGCM estimates by sex subsample and z-tests of sex differences.

Parameter	Males (n = 1,405) *B* (*SE*)	Females (n = 1,185) *B* (*SE*)	*z*	*p*
	Growth factor means: Smartphone dependence			
Intercept	33.378*** (0.361)	33.671*** (0.378)	−0.56	.575
Pre-COVID slope	1.712*** (0.218)	1.175*** (0.223)	1.72	.085†
COVID step	−0.974** (0.355)	−0.444 (0.354)	−1.06	.291
Post-COVID slope	−0.020 (0.076)	−0.312*** (0.074)	2.75	.006**
	Growth factor means/intercepts: Sleep quality			
Intercept	3.758*** (0.118)	3.870*** (0.099)	−0.73	.467
Pre-COVID slope	−0.025 (0.027)	0.033 (0.025)	−1.58	.115
COVID step	0.008 (0.036)	−0.002 (0.039)	0.19	.851
Post-COVID slope	−0.056*** (0.008)	−0.039*** (0.009)	−1.41	.158
	Growth factor means/intercepts: Suicidal ideation			
Intercept	1.076*** (0.236)	1.619*** (0.282)	−1.48	.140
Pre-COVID slope	−0.037 (0.034)	−0.168*** (0.041)	2.46	.014*
COVID step	−0.057 (0.040)	0.161*** (0.045)	−3.62	<.001***
Post-COVID slope	0.035*** (0.009)	0.027* (0.011)	0.56	.573
	First-stage mediation path (a): SDep → SQ			
Intercept	−0.019*** (0.003)	−0.022*** (0.003)	0.71	.480
Pre-COVID slope	−0.002 (0.010)	−0.028*** (0.006)	2.23	.026*
Post-COVID slope	−0.023** (0.007)	−0.036*** (0.007)	1.31	.190
	Second-stage mediation path (b): SQ → SI			
Intercept	−0.180** (0.057)	−0.322*** (0.067)	1.61	.107
Pre-COVID slope	−0.633** (0.230)	−0.999 (0.537)	0.63	.531
Post-COVID slope	0.066 (0.080)	0.176 (0.092)	−0.90	.367
	Direct effect (c′): SDep → SI			
Intercept	0.031*** (0.003)	0.024*** (0.003)	1.65	.099†
Pre-COVID slope	0.038** (0.012)	0.029 (0.018)	0.42	.678
Post-COVID slope	0.048*** (0.007)	0.061*** (0.011)	−1.00	.319
	Indirect effect: SDep → SQ → SI			
Intercept	0.003** (0.001)	0.007*** (0.002)	−1.79	.074†
Pre-COVID slope	0.001 (0.006)	0.028 (0.016)	−1.58	.114
Post-COVID slope	−0.002 (0.002)	−0.006 (0.004)	0.89	.371

Note. The parallel piecewise LGCM specified in the primary analysis (Table 5) was re-estimated separately for male (n = 1,405) and female (n = 1,185) subsamples using maximum likelihood with robust standard errors (MLR) and full-information maximum likelihood (FIML). Sex was omitted from within-group models because the sample was already partitioned by sex. Model fit was acceptable for both groups (males: $\chi^2(136) = 382.97$, CFI = .916, TLI = .905, RMSEA = .036 [.032, .040], SRMR = .041; females: $\chi^2(136) = 364.72$, CFI = .939, TLI = .932, RMSEA = .038 [.033, .042], SRMR = .041). Sex differences were tested using z-test for the equality of regression coefficients (Paternoster et al., 1998) computed as $z = \frac{(B_M - B_F)}{\sqrt{SE_M{}^2 + SE_F{}^2}}$, with positive values indicating that the male coefficient is larger than the female coefficient. For endogenous latent variables (sleep quality and suicidal ideation), the reported means correspond to regression intercepts as noted in Table 4. The Heywood constraint on the residual variance of the SI pre-COVID slope (set to 0) was retained in both subsample models to match the primary model. B = unstandardized estimate; SE = robust standard error. SDep = smartphone dependence; SQ = weekday sleep quality; SI = suicidal ideation. Leading zeros for p-values less than 1 are omitted. † $p < .10$; * $p < .05$; ** $p < .01$; *** $p < .001$.
